# Under- treatment and under diagnosis of hypertension: a serious problem in the United Arab Emirates

**DOI:** 10.1186/1471-2261-6-24

**Published:** 2006-06-06

**Authors:** Abdishakur M Abdulle, Nico JD Nagelkerke, Samra Abouchacra, Javed Y Pathan, Abdu Adem, Enyioma N Obineche

**Affiliations:** 1 Department of Internal Medicine, Faculty of Medicine and Health Sciences, United Arab Emirates University, Al-Ain, United Arab Emirates; 2 Department of Community Medicine, Faculty of Medicine and Health Sciences, United Arab Emirates University, Al-Ain, United Arab Emirates; 3 Department of Medical Microbiology, University of Manitoba, Winnipeg, Canada; 4 Department of Nephrology, Tawam Hospital, Al-Ain, United Arab Emirates; 5 Department of Pharmacology, Faculty of Medicine and Health Sciences, United Arab Emirates University, Al-Ain, United Arab Emirates

## Abstract

**Background:**

Hypertension, notably untreated or uncontrolled, is a major risk factor for cardiovascular diseases (CVD) morbidity and mortality. In countries in transition, little is known about the epidemiology of hypertension, and its biochemical correlates. This study was carried out in Al Ain, United Arab Emirates, to characterize self-reported (SR) normotensives and hypertensives in terms of actual hypertension status, demographic variables, CVD risk factors, treatment, and sequalae.

**Methods:**

A sample, stratified by SR hypertensive status, of 349 SR hypertensives (Mean age ± SD; 50.8 ± 9.2 yrs; Male: 226) and 640 SR normotensives (42.9 ± 9.3 yrs, Male: 444) among nationals and expatriates was used. Hypertensives and normotensive subjects were recruited from various outpatient clinics and government organizations in Al-Ain city, United Arab Emirates (UAE) respectively. Anthropometric and demographic variables were measured by conventional methods.

**Results:**

Both under-diagnosis of hypertension (33%) and under-treatment (76%) were common. Characteristics of undiagnosed hypertensives were intermediate between normotensives and SR hypertensives. Under-diagnosis of hypertension was more common among foreigners than among nationals. Risk factors for CVD were more prevalent among SR hypertensives. Obesity, lack of exercise and smoking were found as major risk factors for CVD among hypertensives in this population.

**Conclusion:**

Hypertension, even severe, is commonly under-diagnosed and under-treated in the UAE. Preventive strategies, better diagnosis and proper treatment compliance should be emphasized to reduce incidence of CVD in this population.

## Background

Hypertension, notably untreated or poorly controlled, is a major risk factor for cardiovascular diseases (CVD) in almost every population [1-4]. Although, often controllable by pharmacotherapy [5,6], many patients are unaware of their condition, and thus are untreated, both in Western societies such as the USA [2,7], but particularly among populations in transition [8-10]. Also, many individuals who are aware of their condition are not adequately treated [11]. Many factors contribute to these problems including poor treatment compliance, lack of access to health care and lack of physician adherence to therapeutic guidelines [2,3,5]. The high prevalence of undiagnosed hypertension is emphasized by the large number of cases discovered incidentally e.g. during surveys, or when patients are under going treatment for other diseases, especially in developing countries [12,13].

It appears that the average blood pressure (BP) level and its age-related increase differs across populations and may explain much of the observed disparities in stroke mortality [14]. To date the bulk of the available knowledge about hypertension comes from people of Caucasian origin in developed countries [15]. Little information is available for populations in transition, some of which may have a particularly high genetic risk for the development of diseases of affluence, e.g. the "thrifty gene" hypothesis for diabetes [16,17].

A specific example of a nation in transition is the United Arab Emirates (UAE). As late as the 1960s the population consisted largely of nomadic Bedouin Arabs. However, the discovery of oil in 1970s has changed their life style dramatically and the UAE is now a modern, wealthy society, heavily influenced by Western living patterns, including a sedentary life style with high CVD risk profiles [18]. Indeed, CVDs are known to be the leading causes of morbidity and mortality in the UAE among both the nationals and the expatriates, who constitute the majority (approximately 80%) of the population [19]. Of particular concern is the prevalence of obesity, which reaches 35% among young adolescent Bedouin Arab women [20]; and about 24% among medical students [21]. These students also reported high stress levels (65%), unhealthy diets (50%), and low levels of physical activity (77%) – Perhaps attributable to cultural and climatic restrictions [22]. Also, smoking has increased among men [23-25]. Hypertension is also common with prevalence reported as 19–25% [26], and 15.3% (urban populations) and 10.6% (rural populations) [27]. However, in these studies no information was collected from the expatriate communities. Neither did they obtain any data on treatment and treatment compliance.

The present study specifically addresses the characteristics of self reported hypertensives, and self reported normotensives in terms of demographic variables, the prevalence of life-style risk factors, under-diagnosis, and treatment outcome.

## Methods

The study was approved by the Ethics Committee of the Faculty of Medicine and Health Sciences, United Arab Emirates University, Al Ain, UAE.

Subjects were enrolled over the period February 2001 to May 2005. The sample was stratified by SR hypertension (yes/no) and consisted of 349 SR hypertensives, and 640 SR normotensives (controls) among Arab nationals and expatriates (non-Gulf Arabs, Africans, Asians, Europeans and Far East Asians). There were 670 males and 319 females. Hypertensive subjects were recruited from various outpatients' clinics in Al-Ain city, UAE. Controls were enrolled from among University staff, teachers of secondary schools, factory workers, and various other institutions, to obtain a broad and representative cross-section of the adult population of Al-Ain.

Individuals between the age of 20 and 75 years with SR hypertension were referred to a special project physician in the hypertension clinic in Tawam Hospital, Al-Ain, to exclude secondary hypertension and known kidney disease or heart problems (no patients, however, had to be excluded for these conditions). Both patients and controls were then informed, in either Arabic or English, about the project's objectives and methods. Informed, written consent was obtained in all cases. Each individual was interviewed by a healthcare professional, using a one-page questionnaire, in Arabic or English, regarding demographic variables including ethnic background (country of origin), history of hypertension, diabetes, history of dyslipidaemia, current smoking (smoking cigarettes every day), current exercise (exercising at least for one hour/three times per week), and current alcohol intake (drinking alcohol at least once/three times per week). The questionnaire was piloted among primary health care patients and adapted accordingly. Although all patients had been prescribed anti-hypertensive treatment, no details on the exact medication were elicited. Besides to their prescribed conventional anti hypertensive drugs, many SR hypertensives were on lipid-lowering treatment if they had concomitant diabetes or dyslipidaemia.

All subjects were instructed to relax in a sitting position for at least 15 minutes prior to BP measurements. A mercury sphygmomanometer with appropriate cuff size was used for BP measurements. A specially trained nurse took two BP measurements for each individual in two different positions (sitting and standing) the average of which was used for analyses. Individuals with high BP levels among the SR normotensive group (incident of hypertension) were referred to a physician.

Hypertension was defined according to the Seventh Report of the Joint National Committee on Prevention, Detection, Evaluation, and Treatment of High Blood Pressure (JNC-7) definition [3]: normal (systolic < 120 mm Hg and diastolic < 80 mm Hg), pre-hypertension (systolic = 120–139 mm Hg or diastolic = 80–89 mm Hg), stage I (systolic = 140–159 mm Hg or diastolic = 90–99 mm Hg), stage II (systolic ≥ 160 mm Hg or diastolic ≥ 100 mm Hg). Incident (new, undiagnosed) hypertensives were defined as control subjects with a systolic blood pressure (SBP) ≥ 140 mm Hg and/or a diastolic blood pressure (DBP) ≥ 90 mm Hg. Under-treated hypertensives were defined as patients having BP levels in the above mentioned range.

In this study, weight, height, waist and hip circumference were all measured according to the WHO MONICA protocol [28]. Body mass index (BMI) was calculated as weight/height^2 ^(kg/m^2^), and waist to hip ratio (WHR) was also calculated. Obesity was defined as BMI ≥ 30 kg/m^2 ^and central body fat distribution was defined as a WHR ≥ 0.9 for men and ≥ 0.8 for women. These variables were measured by trained staff nurses in either the outpatient hypertension clinic in the case of hypertensives or in a special room in other organizations. Measurements were taken without shoes, and no heavy clothing was worn at the time of the investigations.

### Statistical analysis

For statistical analysis of the total data set, the Statistical Package for the Social Sciences (SPSS) Version 13.0 for Windows (SPSS, Chicago, U.S.A.) was used. Associations between categorical (nominal) variables were analyzed using cross tabulations and Chi-square tests, and the strength of the association was expressed in terms of Odds Ratios (OR), adjustment for confounding variables was carried out using the Mantel-Haenszel estimate of the OR. Continuous variables are presented as means +/- SD. Differences, adjusted for sex, among groups in continuous variables were analyzed using Analysis of Variance, and the Tukey method was used for post-hoc tests (i.e. p-values adjusted for multiple comparisons). P-values < 0.05 were considered statistically significant.

## Results

The number of individuals in each of the three BP groups (SR normotensives with normal blood pressure, incident hypertensives, [i.e. SR normotensives who actually had hypertension], and SR hypertensives) broken down by sex and nationality is shown in Table 1. For each of these groups, the prevalence of regular exercise, smoking and alcohol consumption are also shown. The level of under-diagnosis of hypertension (216/640; 33%) and under-treatment (266/349; 76%) were very high. Many SR hypertensives even had blood pressures in the stage II hypertensive range (Table 2). Interestingly, Emiratis, as compared to the immigrants (combined), had a much higher likelihood of having their hypertension diagnosed (Mantel-Haenszel OR, adjusted for sex 2.56 (95% CI: 1.65–3.97).

**Table 1:** Demographic data by blood pressure group and by nationality and gender.

**Ethnicity**	** Numbers (N)**	**Blood pressure groups**					
	Life style risk factors	Normotensives		SR Hypertensives		Incident Hypertensives	
		
		M	F	M	F	M	F
	
Emirates	N	36	34	56	60	20	13
	Regular Exercise (%)	36	44	32	38	80	77
	Cigarette Smoking (%)	3	0	18	2	25	0
	Alcohol Consumption (%)	3	0	5	3	5	0
Non Gulf Arabs	N	82	33	29	12	48	22
	Regular Exercise (%)	60	75	48	50	56	59
	Cigarette Smoking (%)	22	13	24	0	27	0
	Alcohol Consumption (%)	0	9	3	0	0	0
Africans	N	44	10	54	15	22	6
	Regular Exercise (%)	73	80	63	33	68	67
	Cigarette Smoking (%)	20	0	20	0	14	0
	Alcohol Consumption (%)	2	0	2	0	9	0
Asians	N	109	52	82	29	61	9
	Regular Exercise (%)	68	65	57	41	67	56
	Cigarette Smoking (%)	18	0	17	0	21	0
	Alcohol Consumption (%)	17	0	17	3	23	0
Caucasians	N	10	9	1	2	10	3
	Regular Exercise (%)	90	100	0	50	100	100
	Cigarette Smoking (%)	20	22	100	0	0	33
	Alcohol Consumption (%)	90	67	100	50	100	33
Far East Asians	N	0	5	4	5	2	0
	Regular Exercise (%)	0	80	75	60	100	0
	Cigarette Smoking (%)	0	0	25	20	0	0
	Alcohol Consumption (%)	0	20	25	40	50	0

Note: Incident hypertensives are self-reported normotensives with high blood pressure.

**Table 2:** Blood pressure categories according to the JNC seventh report^3^.

			SR Normotensives		SR Hypertensives	
BP Category	SBP (mmHg)	DBP (mmHg)	Female (%)	Male (%)	Female (%)	Male (%)

Normal	< 130	< 85	59.7	45.0	8.1	13.4
Pre-hypertension	130 – 139	85 – 89	13.3	18.2	17.9	8.5
Stage I	140 – 159	90 – 99	24.0	30.4	40.7	37.5
Stage II	≥160	≥100	3.1	6.3	33.3	40.6

Total (N)			196	444	123	224

BP: blood pressure; SBP: systolic blood pressure; DBP: diastolic blood pressure; N: numbers; SR: self-reported. Incident Hypertensives are SR normotensives with high blood pressure.

Table 3 shows the physical measurements of the three BP groups, broken down by gender. These results clearly show a pattern of high levels of CVD risk, with age, BMI and WHR generally increasing from the normotensive group, via the incident hypertensive group, to the SR hypertensives group.

**Table 3:** Demographic and anthropometric characteristics of the three blood pressure groups, by sex.

	**Normotensives **(1)				**P Value **(1 vs. 2)	**Incident Hypertensives **(2)				**P Value **(1 vs. 3)	**Self-Reported Hypertensives **(3)				**P Value **(2 vs. 3)
	F (n = 142)		M (n = 281)			F (n = 53)		M (n = 163)			F (n = 123)		M (n = 226)		

	Mean	SD	Mean	SD		Mean	SD	Mean	SD		Mean	SD	Mean	SD	

Age (yrs)	38.5	8.2	44.2	9.6	0.049	39.7	8.8	45.5	8.2	0.001	49.8	10.3	51.4	8.5	0.001
BMI (Kg/m^2^)	27.8	6.1	26.5	3.7	0.019	29.3	5.4	27.7	5.1	0.000	30.8	6.7	27.9	4.8	NS
WHR (× 100)	84.9	8.7	92.5	5.7	NS	83.4	9.4	92.3	5.7	0.000	91.9	8.6	95.7	4.9	0.000
Waist Circ. (cm)	88.5	13.5	94.8	10.6	.004	92.5	15.6	97.3	12.1	0.000	98.7	12.9	99.2	10.5	0.018
DBP (mm Hg)	77.2	5.5	77.4	5.6	0.000	89.8	6	91.4	7.2	0.000	89.6	10.5	89.8	10.5	NS
Pulse (B/Min)	74.1	7.8	72.9	6.5	NS	75.9	7.8	73.1	7.8	0.030	75.8	9.4	74.2	8.8	NS

BMI: body mass index; WHR: waist to hip ratio; SBP: systolic blood pressure; DBP: diastolic blood pressure; NS: not significant (p > 0.05); Incident Hypertensives are SR normotensives with high blood pressure. The differences between the three methods are tested using Tukey's method, adjusted for sex.

## Discussion

In view of the uncertainty about the pathophysiology of many cardiovascular disorders, their association with life-style in different societies is of particular interest. This was one of the motivating factors for the 7 countries study by Ancel Keys [29]. The rapid life style changes that have taken place in the Gulf Region offer a unique opportunity to study the effect of such changes on CVD morbidity patterns and risk factors.

This study shows that in one of these countries (the UAE), despite a modern and accessible health infra-structure, the levels of both under-diagnosis and under-treatment of hypertension are high, as a large percentage of the SR normotensives and the SR hypertensives actually had BP in the hypertensive range. This situation is similar to that found in many other places in the world, both developed and developing. Even in the USA most hypertension is not properly controlled and among those with untreated or treated but uncontrolled hypertension a majority are aware of their condition [2]. Although our conclusions are based on a convenience sample, our cases appear to be broadly representative for the population of hypertensives in Al Ain, the second largest city in Abu Dhabi Emirate. Although the situation may differ somewhat in the other Emirates, such as Dubai, we do not believe a qualitatively different situation in other parts of the UAE.

The undiagnosed, "incident", hypertensives in out study are in many ways an intermediate group (Table 3), perhaps because the onset of their hypertension is rather recent, which is also suggested by their generally younger age. This may also be due to their better general health than the SR hypertensives, with less concomitant morbidity and symptomatology, and have therefore not been diagnosed as hypertensives before. In addition, a few of them may not be real hypertensives, but only borderline hypertensives, who only occasionally or under special circumstances have BP values in the hypertensive range, and may require regular monitoring.

Among the Emirati nationals, most hypertension had been diagnosed previously and presumably had been treated. It is noteworthy that under-diagnosis of hypertension among non-Emiratis was much more common than among nationals. This may reflect poorer access to health care for immigrants. A large percentage of immigrants are low-income workers, e.g. employed in agriculture or construction. Until recently, the vast majority of such workers had access to (practically) free health care. Currently, the health system is in transition, and it is unclear what the future situation will be. The fact that until recently, their health care had been free, suggests that under-diagnosis is probably not due to financial reasons, but may reflect a different attitude to health and health care among these foreign workers, and perhaps a problem of communication between these workers, who often speak only Urdu or Pashtu, and health care professionals. One possible way to address this situation is to include a more extensive cardiovascular screening component in the periodic health check up (every 2–3 years) that is required for residence permit extensions.

Of special concern is the high level of under-treatment of hypertension. Despite the fact that the SR hypertensives were, by definition, aware of their condition, almost half of them did not have their BP adequately controlled (Table 2). It is unclear whether this is due to inadequate treatment regimens, or lack of compliance e.g. because of asymptomatic nature of hypertension as well as medication side effects. Anecdotically, many individuals diagnosed with hypertension in this region do not properly adhere to treatment, because they partially mistrust the health care providers, and often they have wrong perception of the potential complications from their condition. Clearly, health care providers should better interact with patients to ensure proper compliance. Perhaps, the training of both doctors and nurses should focus more on communicating with patients in this highly multi-cultural environment.

In this population, the problem of under-diagnosis and under-treatment of hypertension is compounded by a high prevalence of other CVD life-style risk factors including smoking, lack of exercise, and obesity. In this context, the levels of obesity, for example, were extremely high with average BMIs in the overweight to obese range (Table 3).

In summary, obesity, lack of exercise and smoking will clearly have a negative impact on the population's health status. Clearly, non- pharmacologic (as well as pharmacologic) approaches to the prevention of CVD among hypertensives and somewhat among normotensives are also needed in this population. The focus therefore should be on prevention, and efforts should address modifiable life style risk factors, such as diet, smoking and lack of exercise. Health authorities in the UAE have recognized smoking of both cigarettes and sheesha (water pipe), as a major health problem and steps have been taken to curb the sale of tobacco to minors and to raise awareness about the dangers of smoking. However, stimulation of regular exercise, known to be associated with substantial reductions in the incidence of coronary events [30], has received less attention, perhaps due to the fact that the extremely hot summer (June – September) constitutes a clear obstacle to outdoor activities. But, even during the pleasant winter months (October – March), few people exercise on regular basis. Overall, it is estimated that slightly over 50% of males, and 39% of females, exercise regularly [31]. There are also other, less clear, obstacles such as cultural perceptions and attitudes, and ad-hoc urban planning marked by lack of community based sport facilities. Despite the fact that some citizens will not change their life style whatever interventions are put in place [32], many others are likely to respond positively to more appealing interventions such as attractive recreational facilities.

## Conclusion

Hypertension in the UAE should be approached more aggressively in terms of prevention strategies, diagnosis and treatment. *Inter alia*, provision of community sports facilities (children play grounds, safe cycling pathways, and indoor swimming pools), adapted to local culture and climate is recommended. Also, there is a pressing need to raise awareness among physicians and patients of the importance of adherence to treatment guidelines and recommendations, and to the potential complications of hypertension.

## Competing interests

The author(s) declare that they have no competing interest.

## Authors' contributions

AA designed the project, collected and interpreted the data, conducted the laboratory analysis and participated in the statistical analysis of the data, and drafted the manuscript.

NN performed the statistical analysis, and further coauthored the manuscript.

SA participated in the patient and control recruitment, carried out clinical examinations for the subjects, and critically reviewed the manuscript.

JP, participated in the measurement of the demographic variables, participated in the recruitment of the subjects and collected blood samples.

AA, participated in the design of the project, and commented on the manuscript.

EO, participated in its design and coordination, provided funds for the project, and critically reviewed the manuscript.

All authors read and approved the final manuscript.

## Pre-publication history

The pre-publication history for this paper can be accessed here:


